# Estimating urban spatial structure based on remote sensing data

**DOI:** 10.1038/s41598-023-36082-8

**Published:** 2023-05-31

**Authors:** Masanobu Kii, Tetsuya Tamaki, Tatsuya Suzuki, Atsuko Nonomura

**Affiliations:** 1 Graduate School of Engineering, Osaka University, 2-1 Yamadaoka, Suita, Osaka 565-0871 Japan; 2 Faculty of Engineering and Design, Kagawa University, 2217-20 Hayashi-Cho, Takamatsu, Kagawa 761-0396 Japan

**Keywords:** Socioeconomic scenarios, Sustainability

## Abstract

Understanding the spatial structure of a city is essential for formulating a spatial strategy for that city. In this study, we propose a method for analyzing the functional spatial structure of cities based on satellite remote sensing data. In this method, we first assume that urban functions consist of residential and central functions, and that these functions are measured by trip attraction by purpose. Next, we develop a model to explain trip attraction using remote sensing data, and estimate trip attraction on a grid basis. Using the estimated trip attraction, we created a contour tree to identify the spatial extent of the city and the hierarchical structure of the central functions of the city. As a result of applying this method to the Tokyo metropolitan area, we found that (1) our method reproduced 84% of urban areas and 94% of non-urban areas defined by the government, (2) our method extracted 848 urban centers, and their size distribution followed a Pareto distribution, and (3) the top-ranking urban centers were consistent with the districts defined in the master plans for the metropolitan area. Based on the results, we discussed the applicability of our method to urban structure analysis.

## Introduction

Understanding the spatial structure of a city is essential for formulating a spatial strategy for that city. For this reason, many city officials and planners devote considerable resources to maintaining accurate data on the cities’ geographic features. Perhaps the two most crucial features are the spatial extent of the city and the layout of the centers of people’s activities. Classical urban economic models describe a mechanism by which transportation costs and land rents determine the extent and density of a city under a monocentric structure^1^; however, many large cities have expanded and developed to have multiple urban centers because of population growth and advances in transportation technology^2,3^. Beyond their relevance to urban planning and governance, the extent of a city and the location of its urban centers have a significant effect on the lives of citizens—through their choice of residence and daily commute—in addition to disaster resilience^4,5^; the peri-urban ecosystem and natural environment^6,7^; and, more recently, infectious diseases^8,9^.

For this reason, various methods for quantitatively analyzing the spatial structure of cities have been explored. Perhaps the simplest strategy is to identify urban areas and population centers by analyzing various forms of statistical data, such as spatial distributions of population and employment^10^, commuting and shopping traffic^11,12^, or activity density and concentration^13–18^. However, the use of statistical data has disadvantages: spatial units of data aggregation and observation frequencies vary from country to country and region to region, and measurements may be spatially coarse and infrequent. To address these challenges, recent studies have investigated methods to characterize urban structure using two alternative data sources: remote sensing data and mobile terminal location data. These data sources provide frequent measurements and high spatial resolution across extensive coverage areas, even in developing nations. Varieties of remote sensing data considered to date include various earth reflectances of the electromagnetic spectrum (see review paper^19^), light detection and ranging^20^, synthetic aperture radar^21,22^, stereoscopic digital surface models (DSMs)^23–26^, and nighttime lights^27–30^. In studies using mobile terminal data, researchers have considered the use of communication traffic data collected by mobile network operators^31^, check-in data for location-based social networks^32^, frequency of call detail records from mobile terminals^33–35^, and Google location histories^36^. However, the location data of cell phones are held by private companies, such as cell phone companies. The data are not disclosed to the public because of privacy protection concerns. By contrast, many remote sensing data are widely disclosed by public organizations.

In one prominent study that captured urban centers in large cities using remote sensing data, Chen et al.^27^ proposed a method to define urban centers using a nighttime light contour tree. They created a contour tree of nighttime lights for Shanghai and successfully detected the city center based on the threshold of nighttime lights. However, Chen et al. (1) defined the hierarchical level of urban centers using contour tree topology and it did not use the light intensity of urban center activity for the systematic activity level evaluation, and (2) set the threshold for urban center detection arbitrarily to match known urban centers that serve as references. The level of activity in city centers is essential information for urban planning and transportation planning; however, Chen et al. did not directly interpret nighttime light intensity in the planning context. They detected 33 urban centers in Shanghai with a population of more than 23 million, which means that they detected only major centers and ignored minor centers by truncating peaks below the threshold or averaging out small peaks.

The definition of urban center is ambiguous^26^. Therefore, various methodologies exist for the identification of polycentricity and subcenters, with different methods used in different studies. For example, Duranton and Puga^37^ suggested that subcenters can range from large to small depending on their levels of functions. To address these issues, we propose a methodology to identify the hierarchical structure of all urban centers based on a contour tree, which reflects the activity intensity of urban centers.

The method proposed in this study is superior to existing methods in three respects. First, it evaluates the spatial distribution of urban activity using a model that transforms remote sensing data into trip attraction. As found by Burger and Meijers^11^, it is straightforward to understand the spatial distribution of urban activities as trip attraction, and to interpret its meaning in urban planning practice. A few studies have been conducted on the relationship between nighttime lights and traffic^38,39^. In this study, we employ statistical modeling to estimate trip attraction using remote sensing data. Specifically, we divide the traffic volume index into two categories: trips going out and trips returning home, based on the purpose of travel. This approach allows us to account for the empirical observation that the attraction volume of trips going out is influenced by the intensity of urban center activities, whereas the attraction volume of trips returning home is influenced by the intensity of residential areas. Thus, we can identify urban centers as the focal points of outgoing trips. We can recognize urban centers as places where going out trips are concentrated. By contrast, we can assume that the destination of a returning home trip is a residential area. Therefore, we can assume that the destinations of these two trips can define urban areas.

In previous studies, most land use and cover data classified land directly based on the surface reflectance spectrum. By incorporating the process of converting remote sensing data into trip attraction volume, we expect to be able to estimate urban areas that are more meaningful from the perspective of urban planning practice than conventional land use data. Using these models, we attempt to determine the spatial extent of the city and the location of city centers.

Second, we extract a comprehensive range of urban centers, from the major centers of the metropolitan area to local community centers, using the contour tree of an estimated going out trip attraction map. In the method of Chen et al., they defined the size and level of urban centers to be extracted using a specific threshold and ignored small centers. Our proposed method is unique in that it extracts a wide range of peaks of the trip attraction map as urban centers. Third, we use the topology information of the contour tree and measure the activity level of the extracted centers by cumulative trip attraction, including their hinterlands. This approach enables us to rank the centers while considering the overall structure of the city. It allows for an analysis that captures the competition among urban centers as well as the independence of suburban centers. This is not achievable when measuring the intensity of activity in urban centers solely based on local conditions, such as a threshold. Taking advantage of these features, in this study, we evolve a method for extracting the urban structure using remote sensing data. As discussed below, the proposed cumulative trip attraction index obtained by expanding the contour tree method achieved higher performance for urban center detection than the ordinary index obtained by the simple contour tree. This is an innovation in this study that advances previous research.

In this study, we use trip attraction as a functional variable of urban structure. We create a model with trip attraction as the dependent variable and morphological variables from remote sensing data as explanatory variables. We use nighttime light data and a DSM as input remote sensing data; however, these data can be replaced depending on the context. The trip attraction volume is statistical data and the unit of aggregation is the traffic analysis zone (TAZ). Generally, TAZs are smaller in the central area than in suburban areas, and TAZs are typically larger than the grid size of remote sensing data. We estimate the model of trip attraction using the data with TAZ as the spatial unit. By inputting grid-based remote sensing data into the estimated model, we can estimate spatially detailed traffic volume indices. We divide trip attraction according to the travel purpose into going out and return trips. We assume that each destination corresponds to a city center and residential area, and model each trip attraction. We use the estimated traffic volume indices to identify urban areas and urban centers. In particular, for urban centers, we replace the input information of the model proposed by Chen et al. from nighttime light with the estimated trip attraction density (TAD) to obtain a hierarchy of urban functions and their locations. Thus, we extract the spatial structure of the city. In the "Methods" section, we explain this analysis procedure in detail.

## Results

### Regression analysis of trip attraction

Before presenting the regression analysis, we check the necessity of the variable transformation of the dependent variable. We tested the parameters of the Box–Cox transformation^40^. The results demonstrated that the parameters were significant at the 1% level for rejecting the null hypothesis of the normality of dependent variable (λ = 1), except for the TAD for return trips with Visible Infrared Imaging Radiometer Suite (VIIRS) nighttime lights (VNL), which was greater than or equal to 50 nW/cm^2^/sr (Table 1). Thus, the TAD for return trips with VNL ≥ 50 was not transformed, and the remainder of the variables were transformed with the parameters shown in Table 1 for the subsequent analysis.

**Table 1:** Box–Cox transformation for dependent variable.

	VNL ≥ 50		VNL < 50	
	Going out	Return	Going out	Return
λ	0.185***	0.901	0.270***	0.375***

Significance: ***p < 0.001, **p < 0.01, *p < 0.05.

To determine the regression model formulated in Eq. (3), we tested all combinations of VNL and altitude difference index (ADI) × VNL as explanatory variables. The details for the ADI are provided in the "Methods" section. We applied the Box–Tidwell transformation^41^ to account for the nonlinearity of the effects of the explanatory variables. We assumed that the transformation parameters were unity if they were not significant. The results are shown in Table 2.

**Table 2:** Estimated model parameters.

		VNL ≥ 50					
		Going out			Return		
		(1)	(2)	(3)	(4)	(5)	(6)
Box-Tidwell	VNL	− 0.804***	–	NA	1.736	–	− 5.690
	VNL × ADI	–	0.450**	NA	–	1.079	0.715
Regression	Intercept	11.23***	3.785***	6.060***	21,533***	18,070***	15,689***
	VNL	− 120.7***	–	− 0.006	− 116***	–	56.1
	VNL × ADI	–	0.181***	0.002***	–	− 6.753***	− 9.065***
R^2^		0.428	0.572	0.556	0.148	0.230	0.238
R^2^ (original)		0.474	0.731	0.700	0.148	0.230	0.239
RMSE		44,464	31,268	35,267	6798	6460	6424
F-value		107.9	192.4	89.4	25.0	43.1	22.3
degree of freedom		144	144	143	144	144	143

		VNL < 50					
		Going out			Return		
		(7)	(8)	(9)	(10)	(11)	(12)
Box-Tidwell	VNL	0.396***	–	0.396***	0.395***	–	0.397***
	VNL × ADI	–	− 0.023***	0.340	–	− 0.024***	0.094
Regression	Intercept	0.629***	105***	0.658***	− 3.255***	300***	− 3.082***
	VNL	2.744***	–	2.709***	8.450***	–	8.299***
	VNL × ADI	–	− 107***	0.001	–	− 310***	0.001
R^2^		0.798	0.448	0.798	0.676	0.382	0.676
R^2^ (original)		0.560	0.266	0.563	0.650	0.306	0.650
RMSE		4116	5386	4099	3147	4475	3148
F-value		5954	1223	2980	3147	933.0	1572
degree of freedom		1510	1510	1509	1510	1510	1509

Significance: ***p < 0.001, **p < 0.01, *p < 0.05.

The upper part of the table shows the results for zones with average VNL ≥ 50 nW/cm^2^/sr, and the lower part shows the results for zones with average VNL < 50 nW/cm^2^/sr. Additionally, (1)–(3) and (7)–(9) are the estimation results for going out and the remainder are the results for the return trip regression model. The notation "-" indicates that the parameter is not applicable.

First, considering the results of the Box–Tidwell transformations, the NA in model (3) means that the estimates diverged and could not be appropriately estimated. Additionally, all variables in models (4)–(6) and VNL × ADI in models (9) and (12) were not significant. We assumed that the influence of these variables was linear.

Next, considering the regression coefficients, all coefficients were significant at the 0.1% level, except for VNL in models (3) and (6) and VNL × ADI in models (9) and (12). Note that the coefficients of VNL in model (1) and VNL × ADI in model (11) were negative, which reflects the fact that the Box–Tidwell exponential was negative. Considering the significant parameters of the Box–Tidwell transformation and regression analysis, we observed that for VNL < 50 and VNL ≥ 50 going out trips, the higher the value of VNL or VNL × ADI, the higher the TAD. By contrast, in the model for return trips with VNL ≥ 50, the larger the values of VNL or VNL × ADI, the lower the TAD. This reflects the negative correlation between the TAD and the variables in the VNL ≥ 50 zone, as shown in Fig. 10, and indicates that the TAD for return trips was low in the city center because the land use was specialized for business.

We considered R^2^ between the estimated and observed values, where R^2^ denotes the multiple correlation coefficient calculated for the Box–Cox-transformed dependent variable. We calculated R^2^ (original) for the dependent variable after transforming the model estimates with the exponential power of the inverse of the Box–Cox parameter and returning to the original TAD scale. R^2^ of the model with two variables, VNL and VNL × ADI, was naturally the highest, except for going out with VNL ≥ 50. For going out with VNL ≥ 50, R^2^ of model (2) was higher because the Box–Tidwell transformation did not yield a solution in model (3). By contrast, one variable was not significant in any of models (6), (9), and (12) with two variables. R^2^ and RMSE did not differ significantly from the model with only the variable that was considered significant in that model. Based on these results, we used (2) for going out with VNL ≥ 50, (5) for return trip with VNL ≥ 50, (7) for going out trip with VNL < 50, and (10) for return trip with VNL < 50.

**Figure 1:**
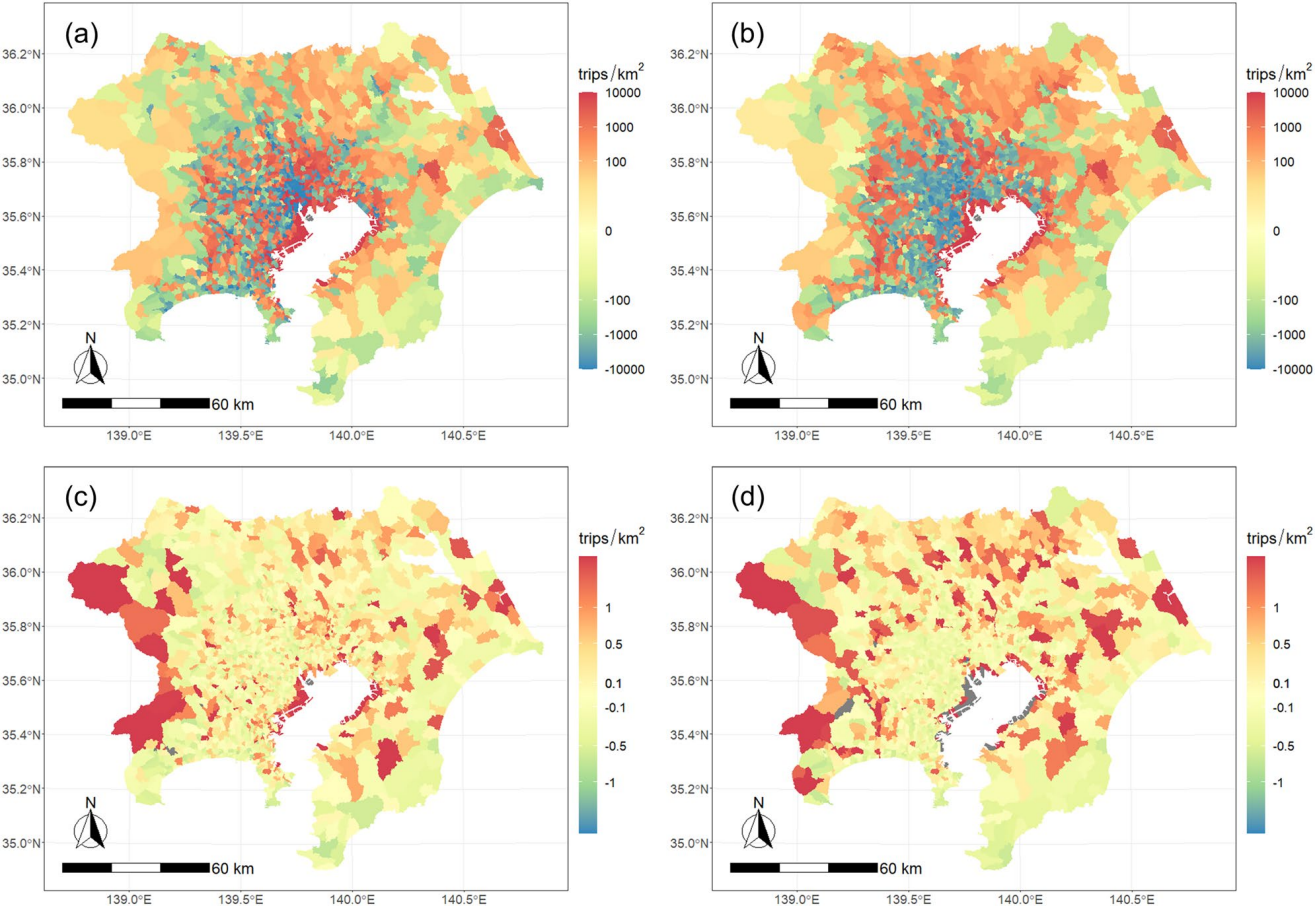
Spatial distribution of the estimation error: (**a**) error of the going out trip, (**b**) error of the return trip, (**c**) relative error of the going out trip, (**d**) relative error of the return trip (the maps were created with the software R 4.1.0^42^ with packages sf 1.0.9^43^, stars 0.6.0^44^, ggplot2 3.4.0^45^, and ggspatial 1.1.7^46^. All maps presented in this paper below were created using the same software.).

The model we obtained above is a simple estimation of the TAD using remote sensing data, but we obtained a certain level of reproducibility. The spatial distribution of estimation errors is shown in Fig. 1. The upper panel of Fig. 1 shows the difference between the estimated and observed TAD, and the lower panel shows the relative error to the observed value. On the left is the going out trip and on the right is the return trip. There was a certain spatial autocorrelation for both going out and return trips. The model estimates were overestimated for the reclaimed areas along the coast of Tokyo Bay because most of these areas are used for the industrial sector. Industrial areas typically exhibit strong nighttime light emissions but tend to have relatively low trip attraction for people. Considering the lower panel, the relative error was larger in less populated zones at the outer edges. By contrast, in densely populated areas, the relative error was rather small. The details of the estimation error for the going out trip in VNL ≥ 50 zones are described in supplementary material S2.

### Urban structure detection on a grid system

**Figure 2:**
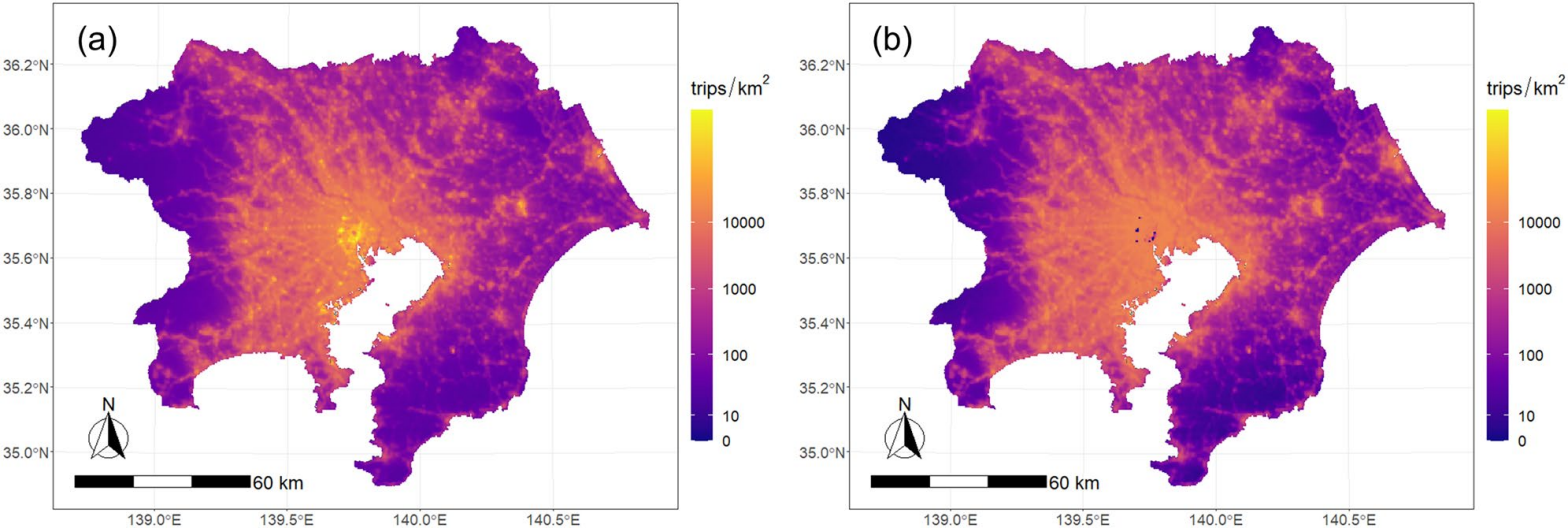
Estimated TAD on the grid: (**a**) going out, (**b**) return.

We applied the above model to grid data to estimate the grid-based TAD. The results are shown in Fig. 2. The figure shows that the overall trend of the target area was the same as that for the zone-based TAD in Fig. 9, but the grid-based TAD provided higher spatial resolution than TAZ-based TAD, particularly in suburban areas.

In the following, we use this estimated grid-based TAD to analyze the extent of the urban area and the spatial distribution of city centers by applying the method described in "Methods" section.

### Estimation of the urban area

**Figure 3:**
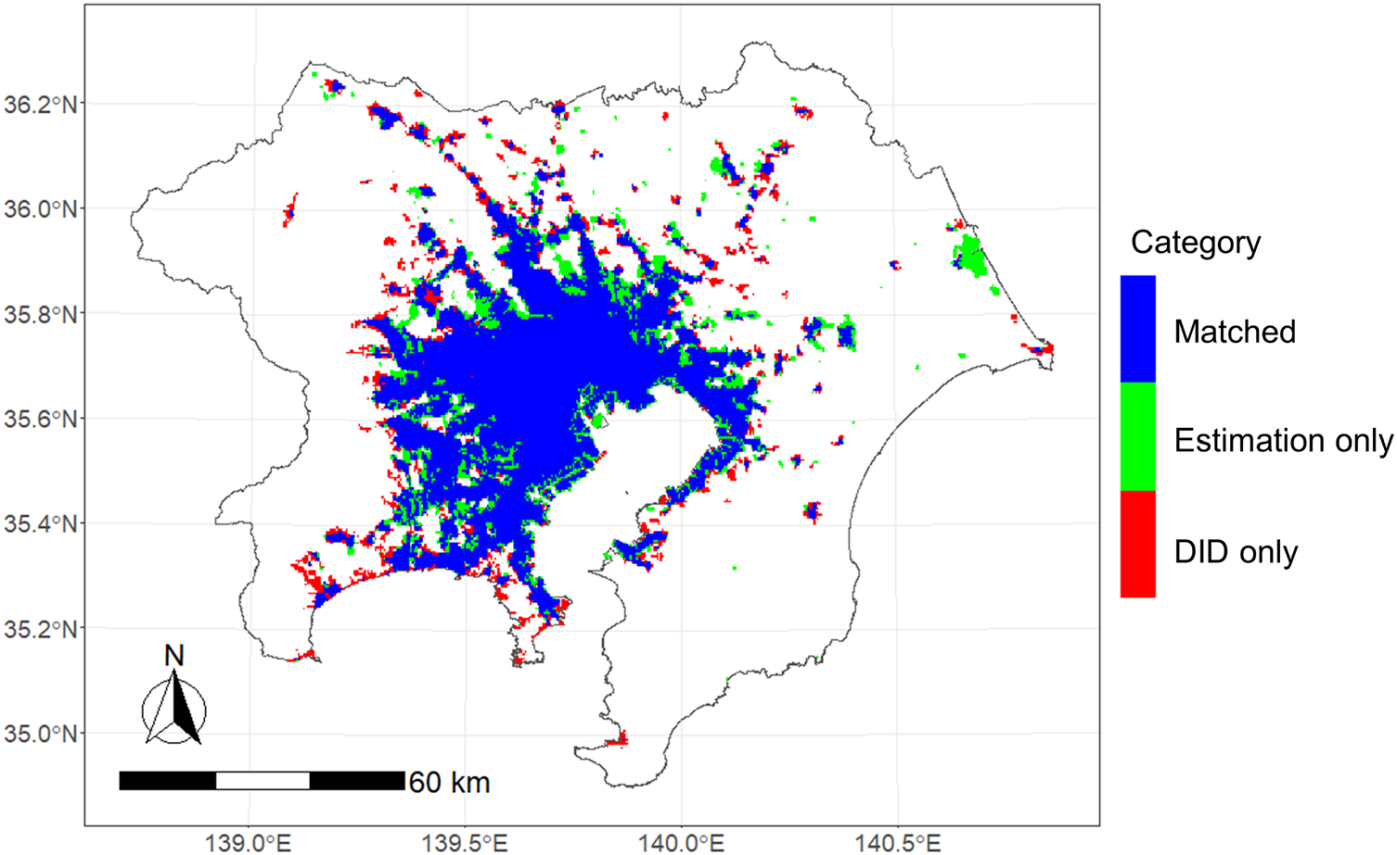
Conformity of the estimated urban area to the DID.

First, we estimated the urban area using Eqs. (4)–(6). We assumed that $f_{u} \left( {q_{Hi} ,q_{Ei} } \right) = wq_{Hi} + \left( {1 - w} \right)q_{Ei}$, and set weight $w$ and threshold $\delta_{M}$ to values that minimize the error from the current urban area. This minimization problem is formulated in Eq. (6). We defined the current urban area as a densely inhabited district (DID), which is a district with a population density of more than 4,000 people/km^2^ and more than 5,000 people in adjacent areas, according to the Japanese census. As a result of the analysis, we estimated the threshold for minimizing the error to be $\delta_{M}$=2722 and the weight to be $w$= 0.461. The fit of the estimated urban area to the DID is shown in Fig. 3.

Figure 3 shows that the estimated area and DID area generally matched in the central area of the metropolis, but there was a large error in the fringe area. In terms of the area, there were 2935 km^2^ of grids where both areas matched, 687 km^2^ of grids where only the estimated area was urban, and 538 km^2^ of grids where only the DID was urban; compared with the total area of the DID, that is, 3474 km^2^, they were 84%, 20%, and 15%, respectively. The DIDs in the periphery were scattered, and remote sensing data-based indices, such as VNL and ADI, were unable to fully capture these urban areas. In particular, grids with a high proportion of natural land use, such as rivers and mountain forests, had a low average nighttime light intensity and were not considered as urban areas by the method. By contrast, there were many highways and large-scale factories in areas that were not DIDs but emitted strong nighttime light and were estimated as urban areas by the method. Although these facilities had a small residential population and did not fall under the category of DID, they were estimated to be urban areas by the method because of their strong nighttime light.

For reference, we compared the urban area defined by DID and that of the ESA CCI Land Cover (CCI-LC) time-series v2.0.7^47^ dataset for 2015 as an example of a conventional method. Regarding the target area, the urban areas in both data coincided in the 3304 km2 grids, but only CCI-LC was urban in the 1972 km2 grids and only DID was urban in the 170 km2 grids. This means that the urban area of CCI-LC was more than twice the urban area of DID. Clearly, the urban areas differ according to their definition. Here, the models used for CCI-LC were not calibrated to represent DID. It is likely that conventional methods would be more suitable for our specific urban areas of interest if the models used for CCI-LC were calibrated accordingly. However, our approach is much simpler than recent sophisticated land use and cover classification methods. We expect it to be relatively easy to calibrate, particularly in urban areas. Further discussion on accuracy is provided in supplementary material S3.

### Estimation of urban centers

**Figure 4:**
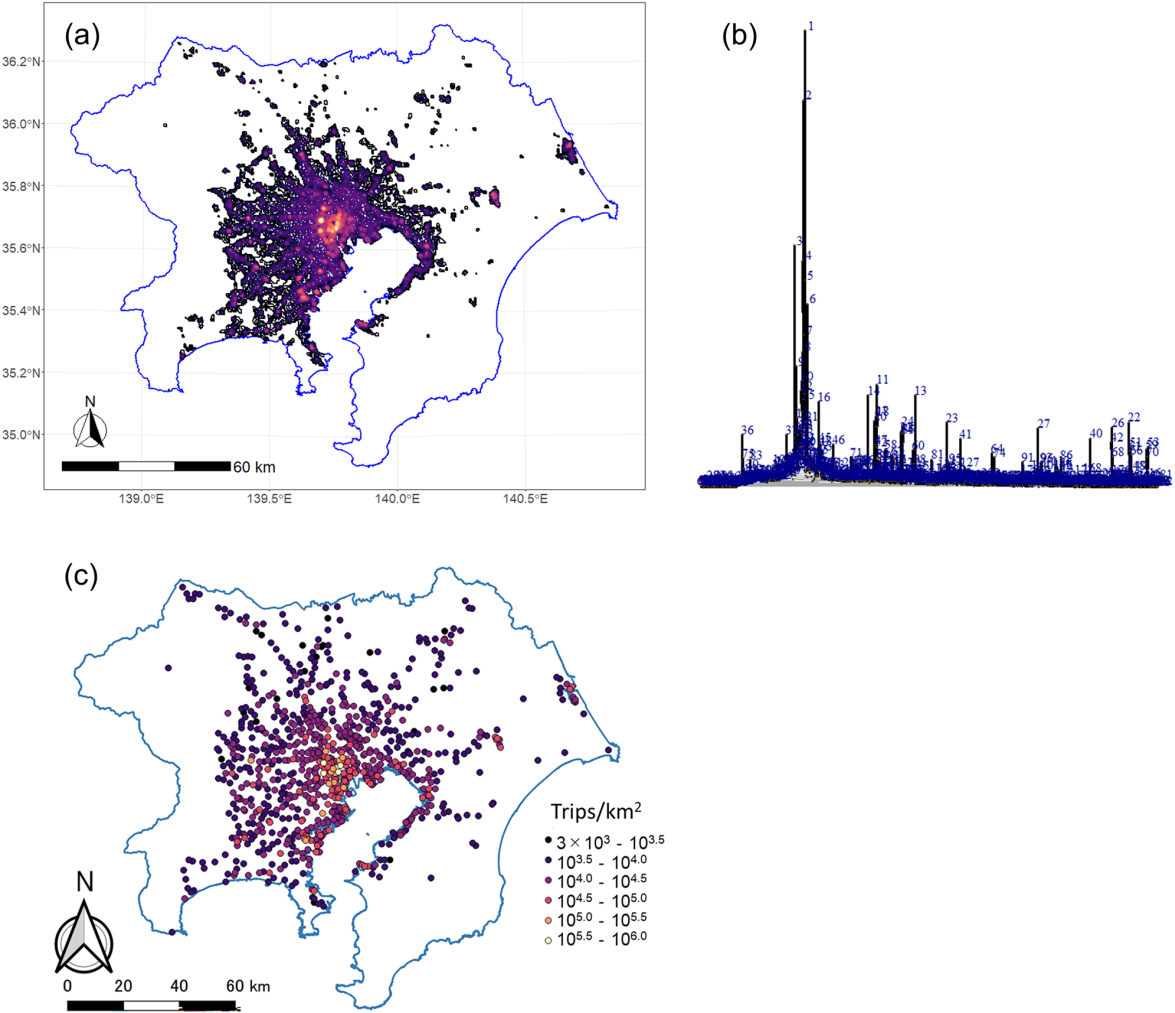
Results of urban center detection: (**a**) contour, (**b**) contour tree, (**c**) seeds.

Next, we extracted the urban centers using the TAD for going out. For grids with a TAD of more than 3,000 trips/km^2^, we created a contour at a level of every 1,000 trips/km^2^, and created a contour tree using the "Methods" described in methods section. The number of contour levels was 653, and the total number of created contours was 7960, of which 848 were seeds. The created contour, its contour tree, and seeds are shown in Fig. 4.

**Figure 5:**
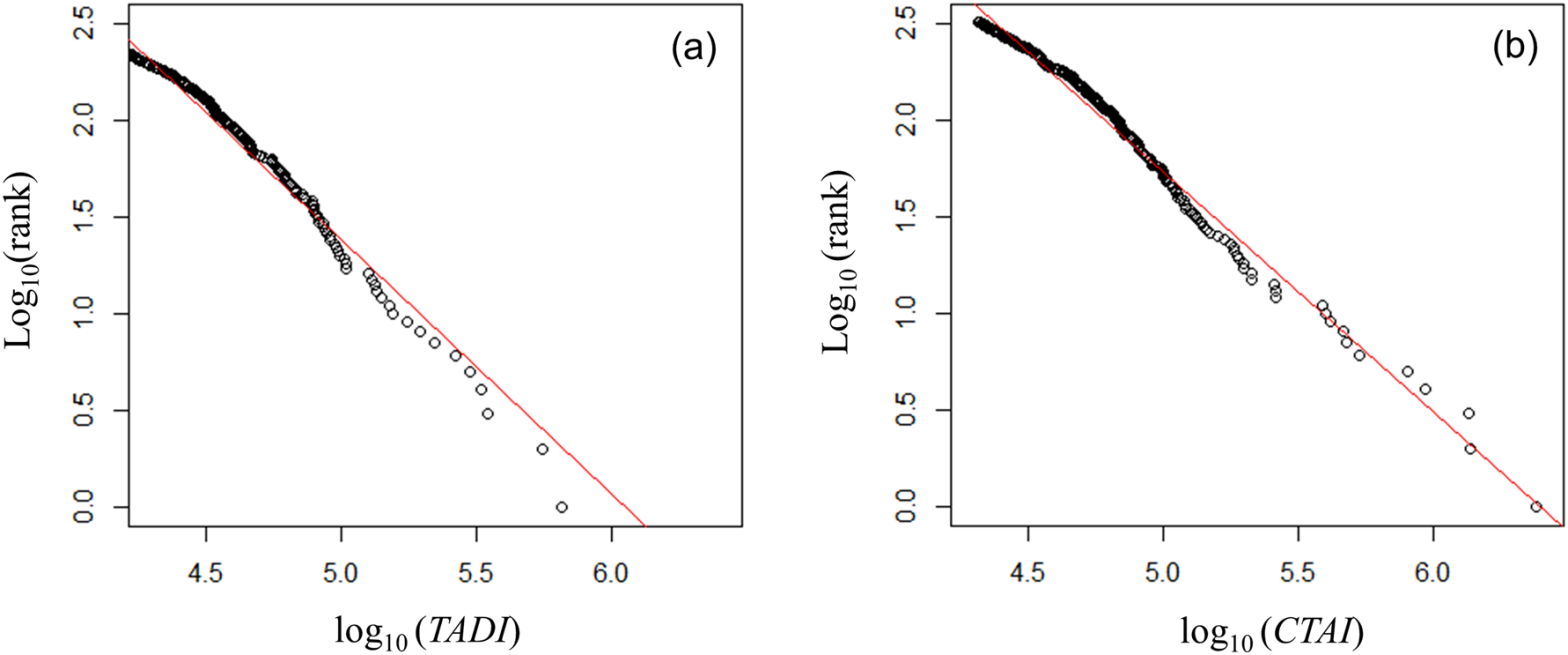
Rank size plot of the trip attraction indices for urban centers: (**a**) TADI, (**b**) CTAI.

We created the contour tree using the "igraph" package v1.2.6 of "R"^48^. We based the layout on the Reingold–Tilford graph layout algorithm^49^, and the height direction pseudo-represents the TAD of each contour. Additionally, in Fig. 4b, we only assigned numbers to the seeds of the contour. The figure shows that seeds with a high TAD are close to each other on the graph, but seeds with a medium TAD are widely distributed, and many seeds have a low TAD. Some urban centers are formed by seeds and their hinterland overall. We evaluated the urban centers using two indices: the original TAD index (TADI) and cumulative trip attraction index (CTAI) at the seed. Cumulative trip attraction is defined in "Methods" section. Figure 5 shows the rank size plot of both indices. The figure shows that both followed a Pareto distribution. We excluded urban centers with fewer than 15,000 trips/km^2^ for the TADI and fewer than 20,700 trips for the CTAI. Therefore, 281 locations are shown for the former and 326 locations for the latter. The values of the Pareto exponent were − 1.32 and − 1.25, respectively. This result implies that the CTAI had a slightly more concentrated distribution than the TADI.

**Figure 6:**
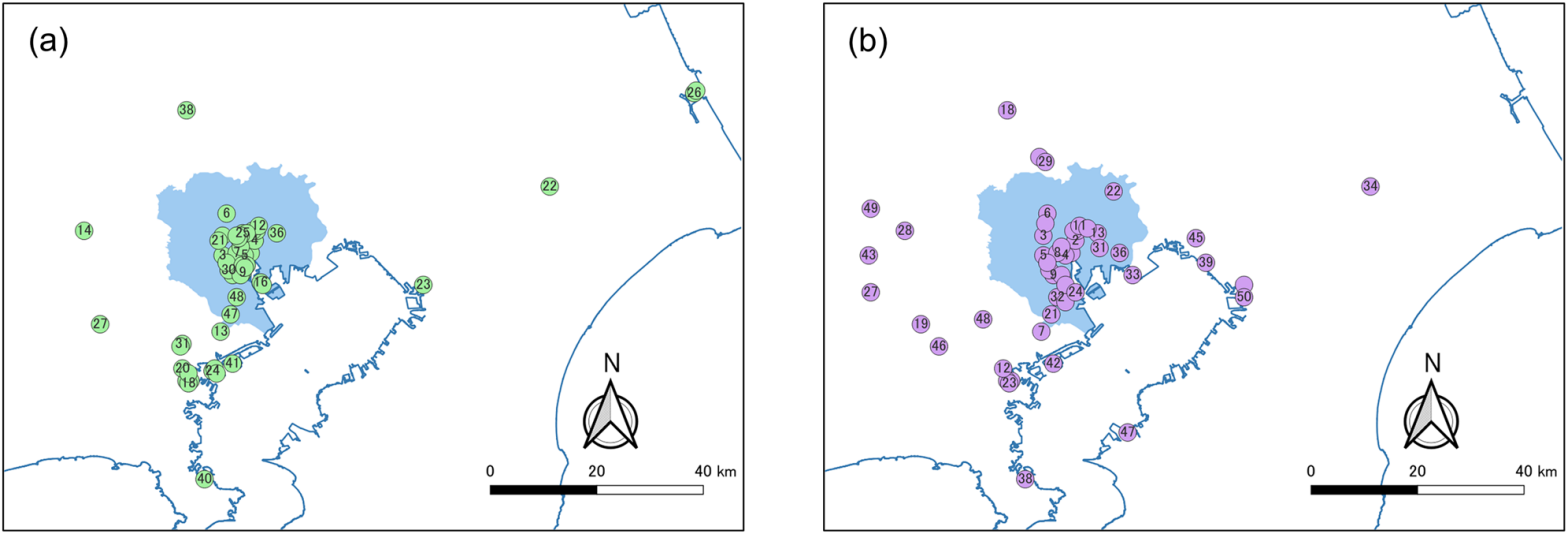
Extracted top 50 urban centers: (**a**) TADI at the seed, (**b**) cumulative trip attraction.

Figure 6 shows the locations of the top 50 urban centers for both indices. The blue zone indicates the 23 wards of Tokyo, that is, the central area of the Tokyo metropolitan area. The figure shows that, although both indices had the largest number of urban centers in the 23 wards, the TADI had a higher concentration of urban centers and fewer urban centers outside the 23 wards. On the other hand, CTAI identified a greater number of urban centers outside the 23 wards.

In the "Guidelines for the Development of the Central Area of the Special Wards of Tokyo" formulated by the Tokyo Metropolitan Government in 1997, the area from the vicinity of Tokyo Station to Shimbashi was designated as the central area of Tokyo. Regarding subcenters, Shinjuku, Shibuya, and Ikebukuro were designated in the National Central Region Development Plan for the Tokyo Metropolitan Area in 1958. Ueno/Asakusa, Kinshicho/Kameido, and Osaki were designated in the Long-Term Plan for the Tokyo Metropolis formulated in 1982. The Tokyo Rinkai subcenter was designated in the Second Long-Term Plan for the Tokyo Metropolis formulated in 1986. In 1986, the National Central Region Basic Plan for the Tokyo metropolitan area called for the development of business core cities on the periphery of the metropolitan area to alleviate congestion problems in city centers. In the current version of the National Central Region Development Plan, Yokohama/Kawasaki, Atsugi, Machida/Sagamihara, Hachioji/Tachikawa/Tama, Ome, Kawagoe, Kumagaya, Saitama, Kasukabe/Koshigaya, Kashiwa, Tsuchiura/Tsukuba/Ushiku, Narita, Chiba, and Kisarazu are designated as business cities to promote the agglomeration of business functions.

**Figure 7:**
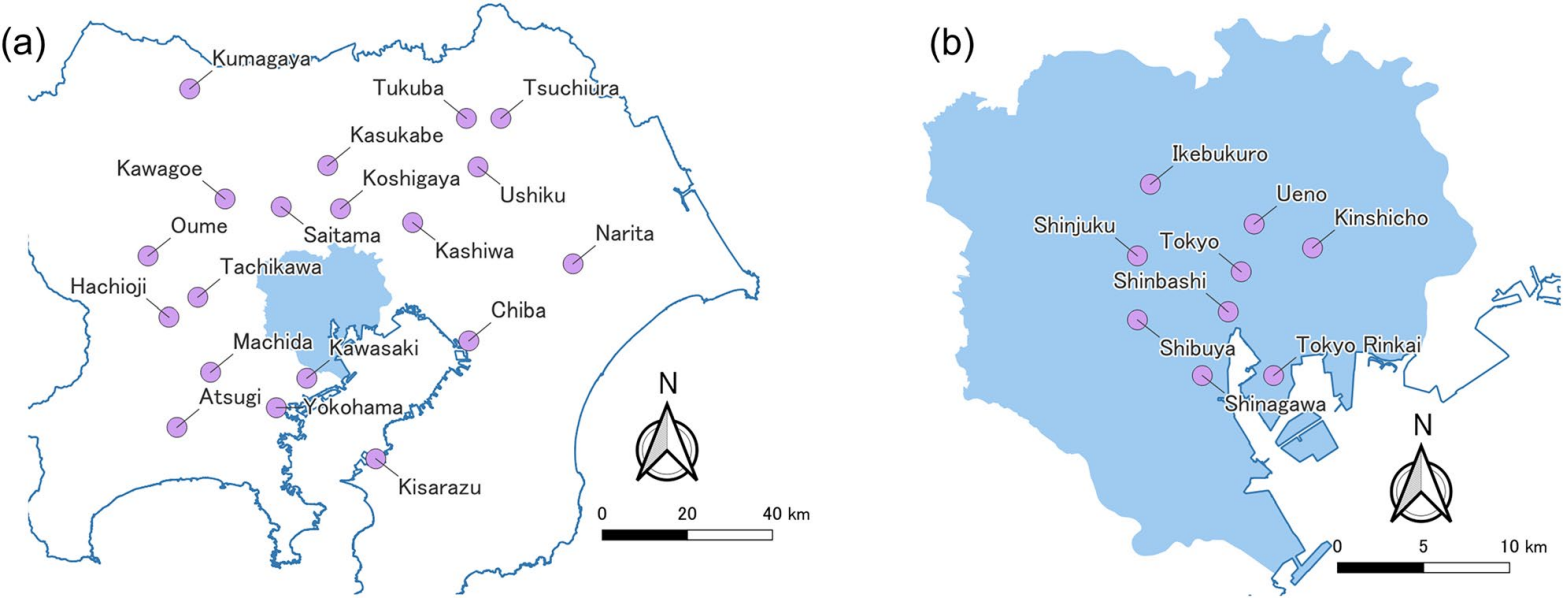
Locations of urban centers in administrative plans: (**a**) centers outside the special wards of Tokyo, (**b**) centers inside the special wards of Tokyo.

The locations of these urban centers are shown in Fig. 7, and the ranks of these urban centers by the two indices are shown in Table 3. Most of the centers in the special wards are ranked within the top 50. By contrast, some of the business core cities are ranked lower than 400th, which suggests that the dispersion of business functions in the plan has not progressed sufficiently. Comparing the ranks of the top centers, in the TADI, Shinjuku, the subcenter, is ranked first, and Shinbashi and Tokyo, the city center, are ranked second and fourth, respectively. In the CTAI, Shinbashi and Tokyo are ranked first and second, respectively, and Shinjuku, the subcenter, is ranked third. This suggests that the CTAI is more suitable for the positioning of urban centers as indicated in administrative plans.

**Table 3:** Rank of urban centers in the master plans for the Tokyo metropolitan area.

Urban centers	TADI	CTAI	Urban centers	TADI	CTAI
Shinbashi	2	1	Atsugi	124	55
Tokyo	4	2	Kashiwa	92	60
Shinjuku	1	3	Tokyo Rinkai	16	77
Shibuya	3	5	Saitama	76	87
Ikebukuro	6	6	Kasukabe	214	90
Kawasaki	13	7	Kawagoe	182	116
Ueno	12	11	Narita	22	123
Yokohama	11	12	Tukuba	547	225
Kinshicho	36	13	Tsuchiura	285	235
Chiba	23	14	Kumagaya	319	246
Machida	27	19	Koshigaya	414	427
Tachikawa	14	28	Oume	551	431
Hachioji	64	43	Ushiku	554	460
Kisarazu	57	47			

The estimation error of the TAD shown in Fig. 1 also affects the generation of the contour tree and subsequent urban center detection. For example, site 41 in Fig. 6a and site 42 in Fig. 6b are at the same location, but they are located in a high-density industrial area and do not attract as many going out trips as a typical urban center. It should be noted that the existence of such an error may lead to the detection of inappropriate urban centers.

Additionally, coastal watchtowers and the lights of highways can be detected as seeds in the contour tree, potentially leading to misidentification as urban centers. In such cases, CTAI may generate low values for isolated seeds since it relies on the cumulative nighttime light within the corresponding contour. To avoid the false detection of isolated nighttime lights as urban centers, it would be effective to filter out low-level CATI seeds. However, it is important to acknowledge that industrial areas and highway interchanges adjacent to urban areas have a higher probability of being detected as urban centers even with CATI. This limitation is inherent in the methodology. To enhance the urban center detection process, it may be necessary to incorporate land use information, road data, and other relevant data sources in conjunction with CATI.

## Discussion

In this paper, we presented grid-based empirical detection of both urban areas and city centers in the Tokyo metropolitan area using remote sensing data that is available worldwide and traffic volume data considering the local context. The result had a higher spatial resolution than TAZ-based activity estimation. Therefore, it can be applied to various types of urban analysis. In this section, we discuss the potential contribution of our method to urban analysis and data challenges for further research.

We represented urban centers as seeds of the contour tree, and evaluated centrality using two indices, TADI and CTAI, for trip attraction. Both indices followed a Pareto distribution, which suggests that the urban center had an agglomeration effect^50^; that is, urban centers with a high trip attraction index tended to attract more traffic. We also demonstrated that the rankings by the CTAI were more consistent with the administrative plan than those by the TADI. This indicates that the identification method of urban centers using the CTAI has high potential to be used as planning information.

The trip attraction indices and the spatial arrangement of urban areas and urban centers estimated by the proposed method can be used for various urban analyses. For example, the going out trip and return trip can be used to estimate trip distribution^51^. The estimated urban centers and urban area can also be used to examine the applicability of central place theory models^52^ and land use simulation models, such as SLEUS^53^. Additionally, it can be used for agent-based traffic simulations^54–56^ because it can estimate the traffic volume on a grid basis, which has higher spatial resolution than TAZ.

The remote sensing data used in this model covers almost all major cities in the world. However, we did not examine the applicability of the trip attraction index model to other cities in this study. Applicability can be examined for cities that have trip attraction data by purpose. However, even in developed countries, the frequency of large-scale travel behavior surveys is approximately once every 10 years at most. Because the nighttime light data used in this paper is continuously observed and published, we believe that the estimation of the spatial structure of the city will be updated in a timely manner using it in a complementary manner with traffic data and other statistics.

By contrast, in developing countries where urban areas are expanding rapidly, large-scale travel behavior surveys are rarely conducted, and information on the spatial structure of urban functions and traffic conditions is insufficient. The proposed model can be used to provide reference information for the spatial structure of urban functions under such data constraints. If we impose the Tokyo parameters on other cities, we can still calculate the urban structure using remote sensing data. However, clearly, there may be considerable bias in that estimation. For example, there is a high correlation between nighttime light and economic activity^57,58^; therefore, applying the model to cities with different levels of economic activity may result in significant bias. Existing land cover data and empirical geographic information of urban centers can be used to examine model applicability and perform calibration. If we can collect appropriate small sample survey data, it might be possible to correct for model bias through calibration. For this calibration, we can apply Bayesian estimation. In the future, we will examine the applicability of the proposed model to other cities with travel data, and compare parameters among cities for meta-analysis. If we have sufficient information about the relationship between remote sensing data and travel behavior data, we may be able to reduce the estimation bias using only remote sensing data. We believe that the proposed approach can contribute to filling the research gap in urban structure estimation using satellite imagery, while taking into account the local context.

At present, numerical elevation data available worldwide are limited in terms of the time of observation and the spatial accuracy of publicly available data are coarse, which makes it difficult to obtain the height of buildings on sloping terrain. Although we limited the estimation error in the target area of this study by combining it with nighttime light, a non-negligible error would arise for cities located on a slope. High-resolution DSMs can provide more accurate estimates of building heights^59^, but such data are not always available for all cities, and availability is on a case-by-case basis. Digital building models in CityGML format are available for some cities (https://3d.bk.tudelft.nl/opendata/opencities/), but are currently limited to major cities in developed countries. To improve accuracy, continuous observation data of building heights must be made available; however, the use of point of interest data^15^ and other data may correct the errors. These issues will be addressed in the future.

## Methods

### Modeling trip attraction using remote sensing data

The *k*th TAZ is denoted by $z_{k}$. Let the concentrated traffic volume be $Q_{k}$ and the area be $A_{k}$. Considering the difference in the areas of TAZs, we estimate a model to calculate the TAD index $q_{k} \left( \lambda \right) = \left( {q_{k}^{\lambda } - 1} \right)/\lambda$, which is the Box–Cox transform of the original TAD ($q_{k} = Q_{k} /A_{k}$). In the case $\lambda = 1$, we assume that $q_{k} \left( \lambda \right) = q_{k}$. We assume that the remote sensing data are given as grid data. Grid $i$ is denoted by $g_{i}$, and the value of remote sensing data *r* is $x_{ri } \left( {i = 1, \ldots ,N_{{{\text{grid}}}} } \right)$. Then, the average values $x_{rk} \left( {k = 1, \ldots ,N_{{{\text{TAZ}}}} } \right)$ at $z_{k}$ are as follows:

1 $$x_{rk} = \left( {\mathop \sum \limits_{{i \in {\Omega }_{rk} }} x_{ri} } \right)/\left| {{\Omega }_{rk} } \right|$$

2 $${\Omega }_{rk} = \left\{ {i{|}g_{i} \cap z_{k} \ne \phi } \right\}.$$

The model that explains the TAD index of the TAZ using remote sensing data is described in general form as follows:

3 $$q_{k} \left( \lambda \right) = f_{q} \left( {\left\{ {x_{rk} } \right\}} \right),$$

where $\left\{ {x_{rk} } \right\}$ means that multiple remote sensing data can be used and $f_{q}$ is a function that converts the value of remote sensing data into trip attraction. We can obtain the model using regression analysis. By applying the grid data to the model $f_{q}$, we can estimate TAD $q_{i}$ of grid $i$:

4 $$q_{i} = f_{q} \left( {\left\{ {x_{ri} } \right\}} \right).$$

We specify $f_{q}$ in the "Results" section. This function and input remote sensing data can be changed according to the local context or the representability of the model.

### Capturing urban structure based on estimated trip attraction

In this study, we capture two aspects of urban structure: urban areas and urban centers. The TADs for going out and return trips at $g_{i}$ are denoted by $q_{Ei}$ and $q_{Hi}$, respectively, which we consider to be correlated with the population density in the daytime and nighttime, respectively. Urban areas are often defined by the population density, whereas urban centers can be captured by activities outside the home.

First, we consider the estimation of urban areas. In this study, the grid set of urban areas is defined as.

5 $$U = \left\{ {g_{i} {|}f_{u} \left( {q_{Hi} ,q_{Ei} } \right) > \delta_{M} } \right\},$$

where $f_{u}$ is the function of the TADs for going out and return trips, for example, $f_{u} \left( {q_{Hi} ,q_{Ei} } \right) = wq_{Hi} + \left( {1 - w} \right)q_{Ei}$, $w$ is weight, and $\delta_{M}$ is the threshold for determining the urban area. Both the composite function and threshold value can vary depending on the regional context. We determine the parameters ($w$, $\delta_{M}$) by solving the following error minimization problem:

6 $$\mathop {\min }\limits_{{w,{ }\delta_{M} }} \left\{ {\left| {U \cap \overline{{U_{b} }} } \right| + \left| {\overline{U} \cap U_{b} } \right|} \right\},$$

where $U_{b}$ is the observed urban grid set, $\overline{U}$ and $\overline{{U_{b} }}$ are the complements of $U$ and $U_{b}$, respectively, and $\left| U \right|$ is the number of elements in set $U$. We specify the function $f_{u} \left( {q_{Hi} ,q_{Ei} } \right)$ and determine parameters ($w$, $\delta_{M}$) in the "Results" section.

**Figure 8:**
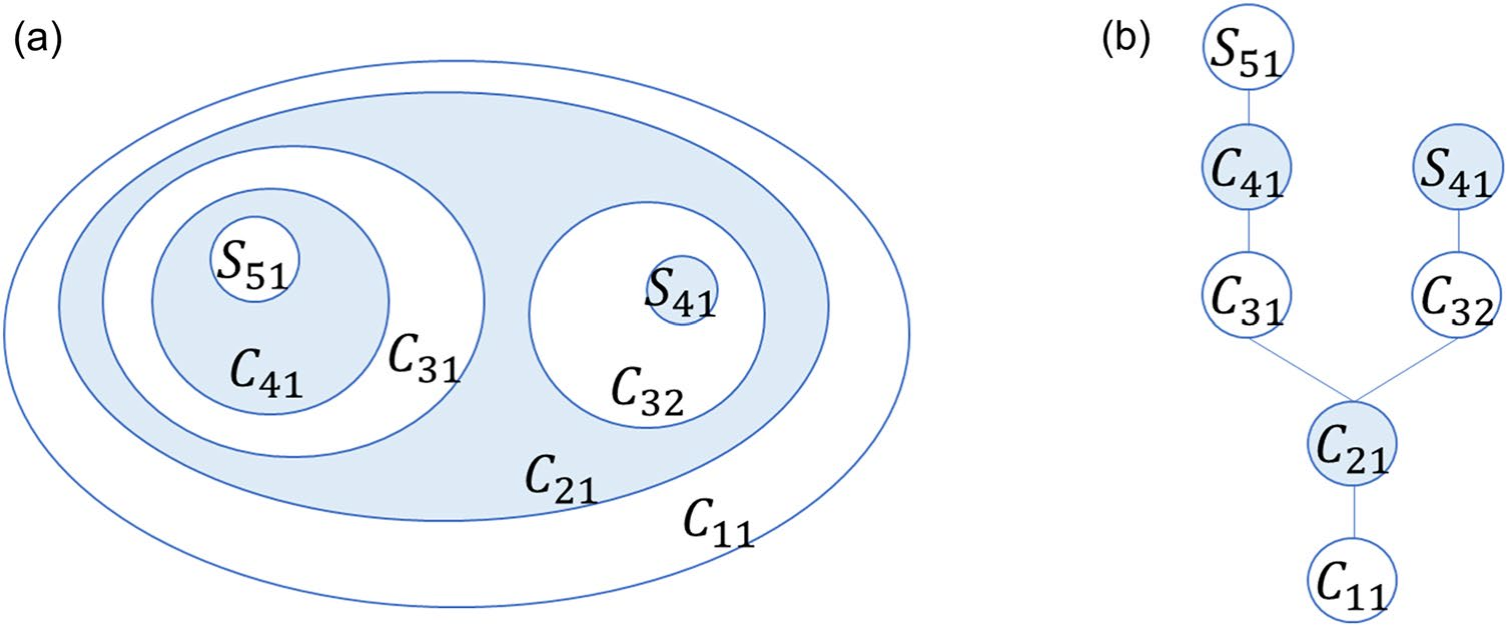
Illustration of a (**a**) contour map and (**b**) contour tree.

Next, because we expect city centers to be the main destination for going out trips, we create a contour tree using the TAD for going out trips. The contour tree consists of nodes and links, where nodes are closed contours and links represent the inclusion relations of closed contours. To create the contour tree, we refer to the method of identifying the hierarchical structure of the space using topographic data^27,60^. First, $C_{hk}$ denotes the grid set contained in the *k*th closed contour of level *h* of the TAD. The $C_{hk}$ for which all the element grids have equal density levels is called the “seed” and denoted by $S_{hk}$; that is, $S_{hk}$ is a local peak grid and set to the endpoint node of the contour tree. If $S_{hk} \subseteq C_{{h - 1,k^{\prime}}}$, then $S_{hk}$ and $C_{{h - 1,k^{\prime}}}$ are connected by a link. Similarly, if $C_{hk} \subseteq C_{{h - 1,k^{\prime}}}$, then $C_{hk}$ and $C_{{h - 1,k^{\prime}}}$ are linked; and if $C_{h - 1,k}$ has links to multiple upper contours, then $C_{h - 1,k}$ is the hinterland of multiple urban centers. Figure 8 shows an example of a contour and contour tree. *C*_11_ contains the only upper node *C*_21_; hence, it is connected by one link in the contour tree. *C*_21_ contains *C*_31_ and *C*_32_; hence, it is connected to the two upper nodes by links. *C*_31_ contains *C*_41_, *C*_41_ contains seed *S*_51_, *C*_32_ contains seed *S*_41_, and *C*_32_ contains seed *S*_51_. Thus, the contour tree represents the inclusion of a closed contour. Hence, we can calculate not only the TAD of the seed but also the traffic volume of any level of the surrounding area that includes the seed to extract the city center district and to analyze the hierarchical structure of city center functions.

Third, we define the cumulative trip attraction at urban centers using the cumulative trip attraction volume of the contour in which each is contained. If there is only one seed in a given contour, we assign the cumulative trip attraction in that contour to the seed, but if there are multiple seeds, we assign the cumulative trip attraction proportionally according to the area of the contour that contains the seed.

Specifically, we follow the steps below. First, the index set of the grid in contour $C_{hk}$ is denoted by ${\Omega }_{hk}^{g} = \left\{ {\forall i;g_{i} \subset C_{hk} } \right\}$ and the index set of the contour at level *h* + 1 in $C_{hk}$ by ${\Omega }_{hk}^{c} = \left\{ {\forall k^{\prime};C_{h + 1,k^{\prime}} \subseteq C_{hk} } \right\}$. If the area of $g_{i}$ is $a_{i}$, the area of $C_{hk}$ and trip attraction are expressed as follows:

7 $$a_{hk}^{c} = \mathop \sum \limits_{{i \in {\Omega }_{hk}^{g} }} a_{i}$$

8 $$Q_{hk} = \mathop \sum \limits_{{i \in {\Omega }_{hk}^{g} }} q_{Ei} \cdot a_{i} ,$$

where $a_{hk}^{c}$ is the area of $C_{hk}$ (superscript *c* denotes the variable for the contour) and $Q_{hk}$ is its trip attraction. If the cumulative trip attraction of $C_{hk}$ is denoted by $Q_{hk}^{m}$, and we assign the cumulative trip attraction up to level *h* of the contour in proportion to the area of the contour within the set ${\Omega }_{hk}^{c}$, and then $Q_{{h + 1k^{\prime}}}^{m}$ is given by

9 $$Q_{{h + 1k^{\prime}}}^{m} = Q_{{h + 1k^{\prime}}} + \frac{{a_{{h + 1k^{\prime}}}^{c} }}{{\mathop \sum \nolimits_{{k^{\prime\prime} \in {\Omega }_{hk}^{c} }} a_{{h + 1k^{\prime\prime}}}^{c} }}\left( {Q_{hk}^{m} - \mathop \sum \limits_{{k^{\prime\prime} \in {\Omega }_{hk}^{c} }} Q_{{h + 1k^{\prime\prime}}} } \right).$$

We assume that $k^{\prime} \in {\Omega }_{hk}^{c}$ and $Q_{1k}^{m} = Q_{1k}$. This trip attraction index takes into account the layout of the city center and the hinterland.

### Target region and data

The target region of this study is the Tokyo metropolitan area. It is the largest metropolitan area in the world, with a population of 36.9 million and an area of 15,950 km^2^ as of 2018. Although several urban centers exist within the region, no single definition of a central district has been set according to local contexts, such as planning and policy.

Trip generation and attraction data by travel purpose by TAZ is available from a person trip survey conducted in the Tokyo metropolitan area in 2018 (https://www.tokyo-pt.jp/special_6th). There are 1,660 TAZs in the person trip survey. In the survey, travel behavior data on a single weekday was collected from September to November in 2018. The sampling rate is approximately 1%. These data provide $q_{k}$ in Eq. (2).

As remote sensing data to describe the traffic volume, we use the Annual product of the Visible Infrared Imaging Radiometer Suite (VIIRS) nighttime lights (VNL) V2^61^ and Advanced Land Observing Satellite World 3D 30 m Resolution DSM (AW3D 30)^62^. The reason for using them is to guarantee the applicability of the method to other cities because they are publicly available for a wide area of the world.

VNL V2 is based on a cloud-free monthly composite generated from the VIIRS Day Night Band, which provides 12-month mean and median values after solar and moonlight reflections and outliers are removed. The grid size is 15 arc seconds and it covers the range from 75 degrees north to 65 degrees south. The content of the record is nighttime light radiance, whose unit is nW/cm^2^/sr. The VIIRS sensor has been in operation from 2012 to the present, and the Annual product of VNL V2 provides yearly data. In the following, nighttime light data are denoted by VNL.

AW3D30 is a set of DSMs released by JAXA in 2016 that were created using images from Panchromatic Remote-sensing Instrument for Stereo Mapping (PRISM) bands aboard the Advanced Land Observing Satellite. PRISM was in operation from 2006 to 2011, and AW3D30 is based on the images taken during that period. The grid size is 1 arc second and it covers the entire land area from 90 degrees north to 90 degrees south latitude. The purpose of this study is to estimate urban structure and we are interested in the height of buildings, therefore we used two methods, the minimum value filter^63^ and slope-dependent filtering technique^24^, to create a digital terrain model (DTM) from the DSM. We created the altitude difference index (ADI) as the difference between the DSM and DTM. For validation, we used the level of detail 1 building data of the 23 wards of Tokyo in PLATEAU (https://www.mlit.go.jp/plateau/), which is a 3D city model. We found that the minimum value filter provided the best fit. We averaged the obtained ADI into the same resolution raster using VNL. In addition, the data acquisition period is an important issue. In the case of Tokyo, the rate of urban development during the period under study is moderate compared with cities in emerging economies, and we assume that it is possible to use the two sets of data together. For more details, refer to supplementary material S1.

**Figure 9:**
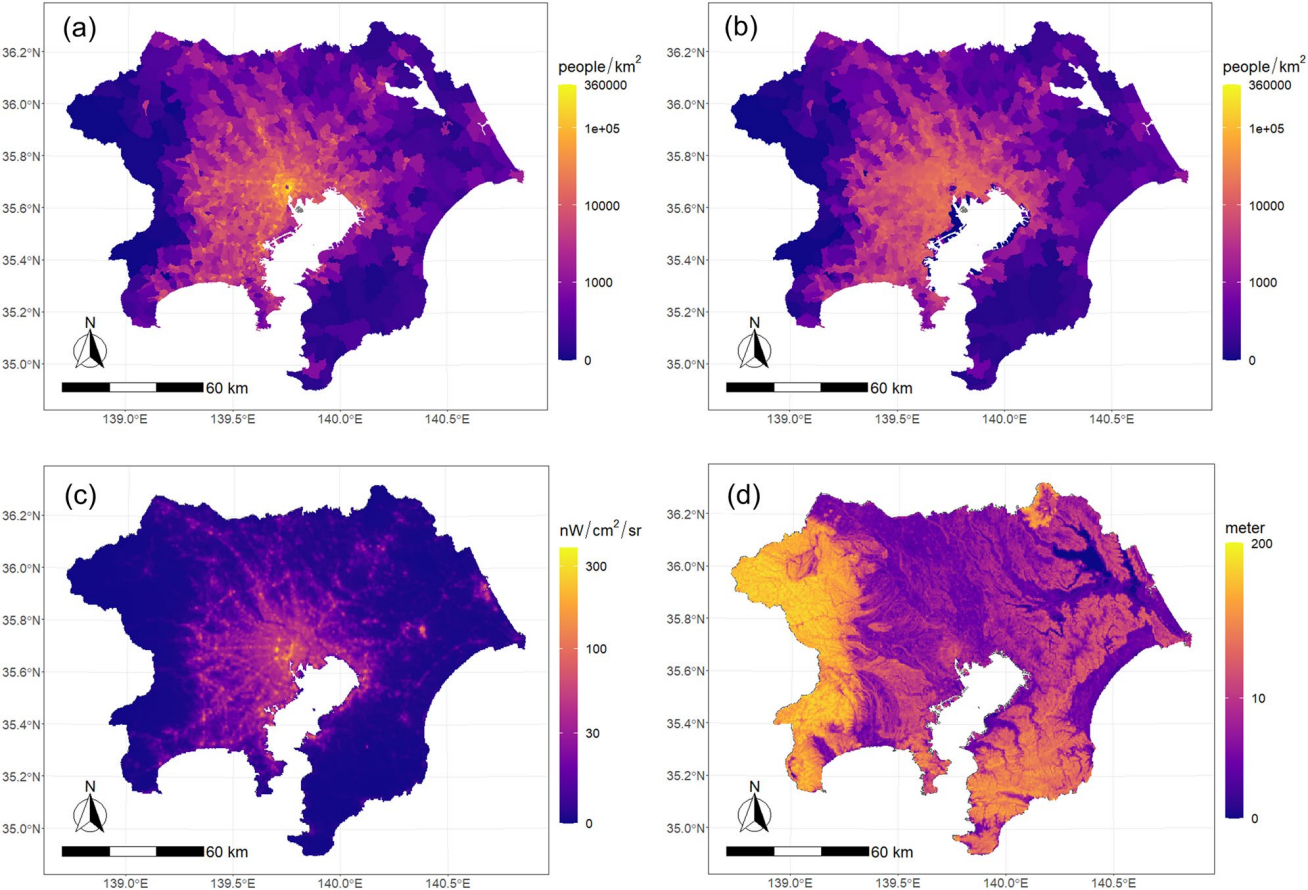
Tokyo metropolitan area data: (**a**) TAD for going out, (**b**) TAD for return, (**c**) VNL, (**d**) ADI.

The TADs for going out and return trips, VNL, and ADI are shown in Fig. 9. The TAD for going out is a high value at the center of the city, which suggests the presence of multiple subcenters. The TAD for return trips tends to decrease from the central area to the suburbs, but the value is low in the central districts and coastal industrial area. VNL is grid data, and may be able to detect the location of subcenters more precisely than the TAZ system. The ADI estimated in this study indicates a higher value in hilly and mountainous areas than in flatland because of the influence of topography, whereas the values in the central part of the urban area are higher, which reflects the height of the buildings.

**Figure 10:**
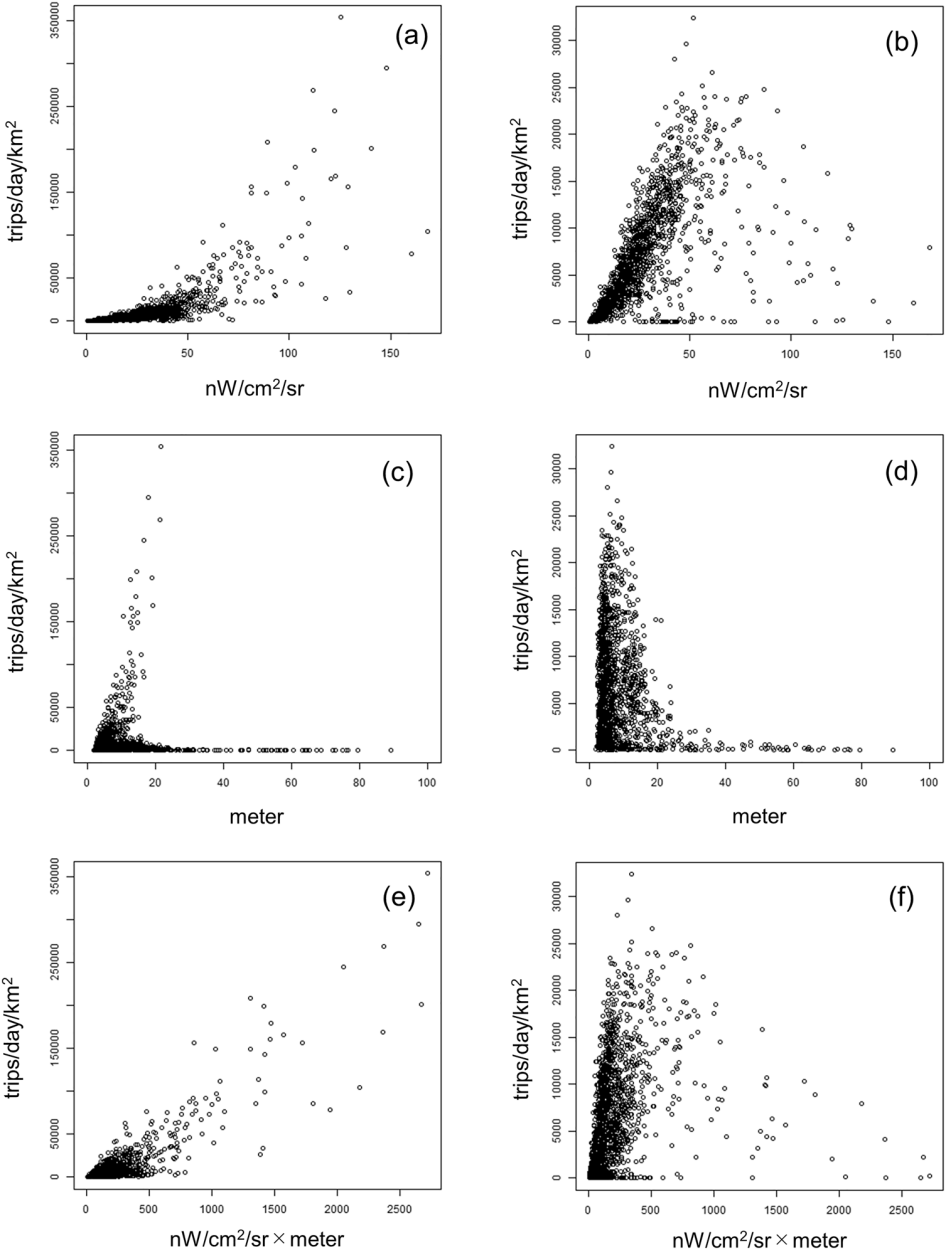
Relationship between trip attraction and indices: (**a**) VNL and going out trip, (**b**) VNL and return trip, (**c**) ADI and going out trip, (**d**) ADI and return trip, (**e**) ADI × VNL and going out trip, (**c**) ADI × VNL and return trip.

Figure 10 is a scatter plot that shows the relationship between VNL, ADI, and product of the two, and the TADs. First, VNL and the TAD for going out are positively correlated, but the variation of the TAD is much larger in the zones where VNL is higher than approximately 50 nW/cm^2^/sr than in the zones where it is lower. Additionally, the TAD for return trips has a strong positive correlation with VNL in the zone below about 50 nW/cm^2^/sr, whereas we observe a moderate negative correlation above that. From both results, we can infer that a zone whose VNL is above 50 nW/cm^2^/sr is a zone in which urban center functions are significant, whereas a zone whose VNL is below 50 nW/cm^2^/sr is a zone in which the main land use is residential. Using a segmented linear regression model^64^, the maximum likelihood estimator of the break point is 45.7 (standard deviation = 1.20) for going out trips and 53.2 (standard deviation = 1.16) for return trips. Therefore, in the following analysis, we classify the region by VNL using 50 nW/cm^2^/sr as the threshold.

Next, we can observe that two types of correlated TAZs and uncorrelated TAZs exist in going out trips. The former indicates that the building volume is high in the zone with a high TAD. The latter results from the fact that the ADI is higher on slopes because of the characteristics of this index. Therefore, it is not appropriate to use the ADI as an explanatory variable for the TAD in a mountainous and hilly area, but it can be used as an explanatory variable for trip attraction in a central urban area. The TAD for return trips does not demonstrate a clear relationship with the ADI.

Considering the relationship between ADI × VNL and TAD, we can observe that the correlation is higher in the zone with a high TAD for the going out trip than in the case of VNL alone; that is, the ADI has the effect of increasing the accuracy of the estimation of the TAD in areas with high VNL, whereas low VNL reflects a low TAD in mountainous zones with a high ADI. Therefore, we can use these two variables in a complementary manner when describing the target region.

Based on the above summary, we consider using the ADI alone as an explanatory variable to be undesirable because of the large error in mountainous zones. Thus, we use VNL and ADI × VNL as explanatory variables in the regression analysis shown in the "Results" section.

## Supplementary Information

Supplementary Information.

## Data Availability

The Annual product of the Visible Infrared Imaging Radiometer Suite (VIIRS) nighttime lights (VNL) V2 was available from the Earth Observation Group website (https://eogdata.mines.edu/products/vnl/). Advanced Land Observing Satellite World 3D 30 m Resolution DSM was obtained from JAXA website (https://www.eorc.jaxa.jp/ALOS/en/dataset/aw3d30/aw3d30_e.htm). Trip generation and attraction data by travel purpose by TAZ was obtained from the 6th Tokyo person trip survey committee website (https://www.tokyo-pt.jp/special_6th). Map for densely inhabited district (DID) was obtained from website of National Land Information Division, National Spatial Planning and Regional Policy Bureau, Ministry of Land, Infrastructure, Transport and Tourism of Japan (https://nlftp.mlit.go.jp/index.html).
