# Supplementary material for: Estimating urban spatial structure based on remote sensing data
Source: Sci Rep. 2023 May 31;13:8804. doi: 10.1038/s41598-023-36082-8 (PMC10232531; doi:10.1038/s41598-023-36082-8)
Supplement: Supplementary file 1 — Supplementary Information. [file 41598_2023_36082_MOESM1_ESM.pdf]

Supplementary Information for:

“Estimating Urban Spatial Structure Based on Remote Sensing Data”

Masanobu Kii, Tetsuya Tamaki, Tatsuya Suzuki, Atsuko Nonomura

S1: Derivation of DTM and ADI

In this study, DTMs were created assuming that the minimum value of 3 to 11 grid squares was the ground surface in the minimum value filter, and Altitude Difference Index (ADI) was obtained as the difference between DSM and DTM. In order to consider the horizontal error, the obtained ADI processed with a 3 to 11 grid-square averaging filter was also validated as DTM. The bare earth grid was obtained using the DTM Filter package of SAGA-GIS (Conrad et al., 2015) as a slope-dependent filtering technique, and the DTM was created by stepwise resampling. The DTM Filter parameters search radius and Approximate Terrain Slope were varied from 1 to 5 and from 0 to 150, respectively. Level of Detail (LOD) 1 building data of the 23 wards of Tokyo from PLATEAU (https://www.mlit.go.jp/plateau/), a 3D city model, was used as the validation data. The data are based on the footprints and heights of 1.77 million buildings in the central Tokyo metropolitan area. Based on this data, the grid average height (GAH), which is the average height of the grid including buildings and the ground surface, was calculated by setting the ground surface height to zero. All the obtained grid data were aggregated in 15 second grid to match the night light data, and the correlation coefficient between ADI and GAH was calculated. As a result, the correlation between ADI and GAH processed by the minimum filter of 3 grid squares and the average filter of 9 grid squares was calculated to be 0.76, which is the highest (Fig. S1). Therefore, the ADI is used as an index of building height in this paper.

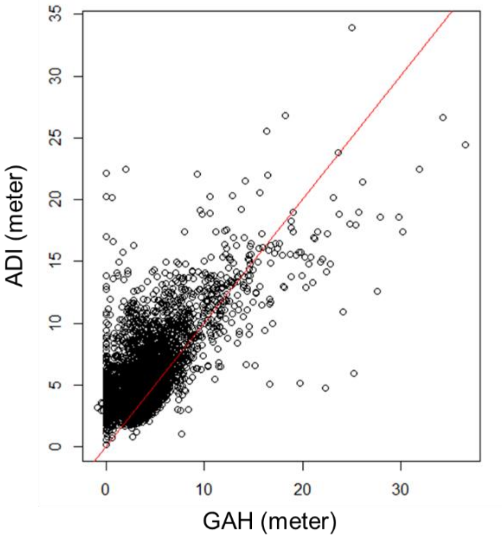

Fig. S1. Scatter plot of GAH and ADI

Fig. S2 shows the GAH and ADI in the 23 wards of Tokyo. GAH based on the 3D city model can represent the volume density of buildings in detail, but the cost of data maintenance is high and the data release is limited to a few cities. The ADI shows that the approximate trend of the height distribution is the same as that of the GAH, but there are some areas that reflect the topography. The higher the resolution of DSM, the higher the accuracy of DTM estimation, and the higher the accuracy of building height estimation is expected to be. If highly accurate building height data is available for the target area, it is desirable to use them.

(a) (b)

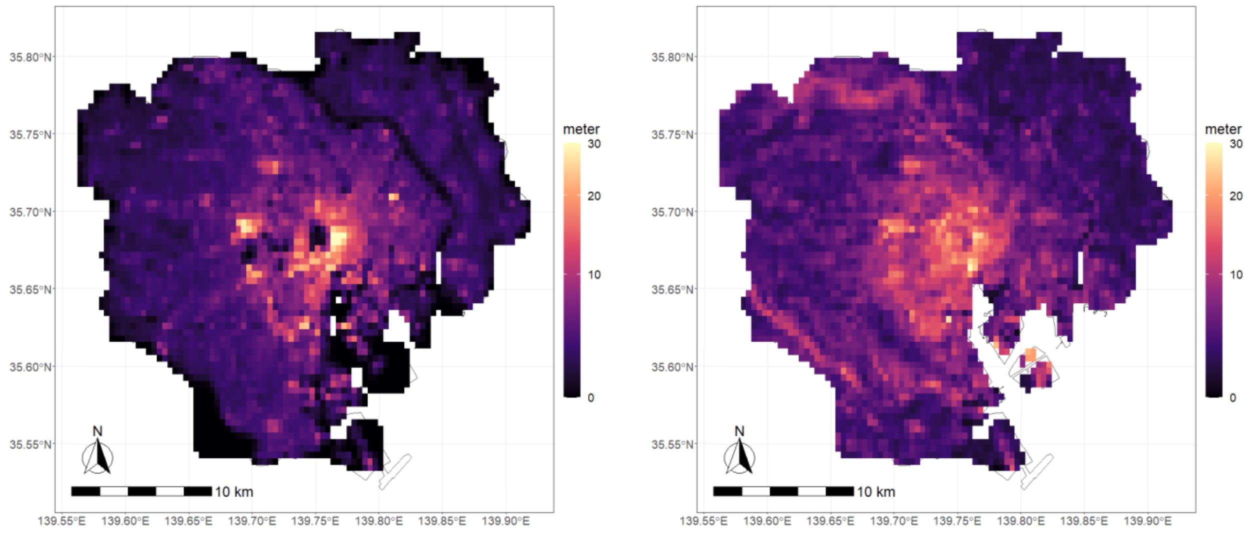

Fig. S2. Height estimation: a) GAH and b) ADI

Conrad, O., Bechtel, B., Bock, M., Dietrich, H., Fischer, E., Gerlitz, L., Wehberg, J., Wichmann, V., & Böhner, J. (2015). System for Automated Geoscientific Analyses (SAGA) v. 2.1.4. *Geoscientific Model Development*, *8*(7), 1991-2007. https://doi.org/10.5194/gmd-8-1991-2015

S2: Estimation error of go-out trip in the zone with VNL≥50

Table 2 shows that for a go-out trip with VNL>=50, there is a difference in the estimation accuracy of trip attraction between the model using only VNL (1) and the model using VNL×ADI (2). To understand its spatial characteristics, the estimation error is plotted on a map in Figure S3. From the figure, it can be seen that the spatial distribution of the error is similar for both models, but the magnitude of the error in the center of Tokyo tends to be relatively smaller for model (2) shown in (b). In particular, the trip attraction of model (1) is overestimated in the eastern area. This area is a mixed area of work and residence, and although the night light is relatively strong, the trip attraction is less than in the area specialized for business. Thus, reflecting the regional characteristics, the model (1) shows a spatial correlation in the errors. VNL will continue to be observed as of 2022, but since DSM is updated infrequently, it is desirable to be able to estimate urban structure using VNL alone, considering future information updates. It should be noted, however, that when estimating using VNL alone, errors due to regional characteristics may occur, as we have seen here.

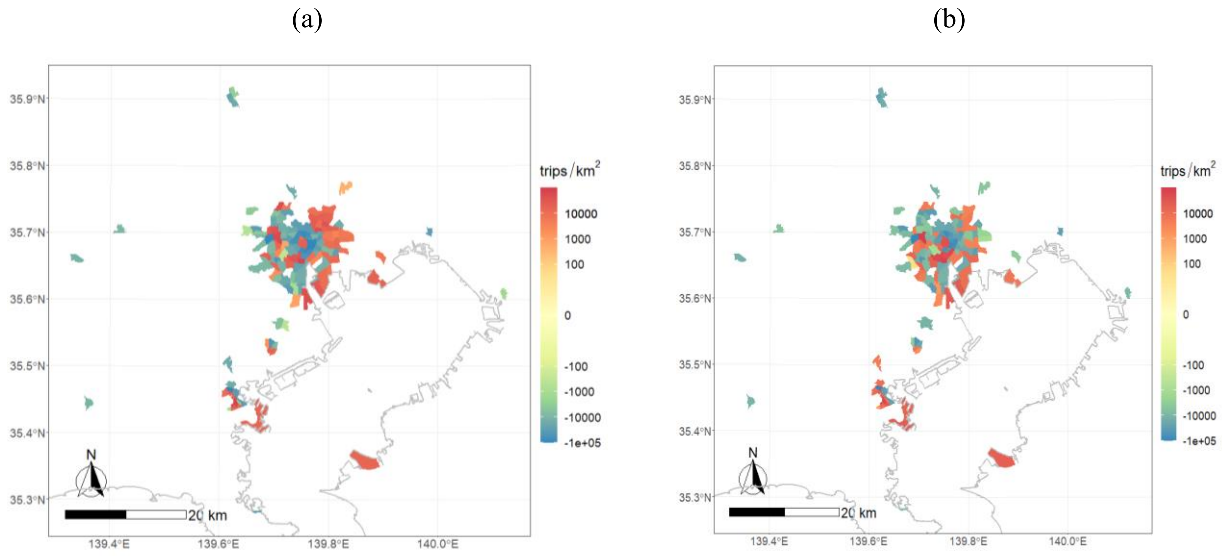

Fig. S3. Go-out trip attraction estimation error: (a) for model (1), and (b) for model (2) in Table 2.

S3: Accuracy of the estimation

Factors affecting estimation error in urban areas

Errors in our estimation of urban areas are mainly found at the urban fringe. Many of the grids that were estimated as urban areas in the model where they were not actually urban areas contained highways and large factories. These facilities have high light intensity such as street lights, but are not administratively defined as urban areas because of the small residential population. If the spatial information of those facilities is given, it may be possible to correct the estimation of this model according to the definition of DID. On the other hand, if the urban area is defined to include these facilities, then the urban area estimated by this model is more representative of the actual situation.

On the other hand, the grids judged as not urban by the model have a large proportion of natural land use, such as rivers, even though they are actually urban areas. In this evaluation, all the grids that intersect the polygons of the DID district are used as urban areas for validation. In other words, many grids with a low percentage of intersecting DID areas are not determined to be urban areas because they have a high percentage of natural land use and therefore low average nighttime light, although they are urban areas in the validation data. Therefore, the estimation error also depends on the definition of the urban area in the validation data. If the urban area of the validation data is determined according to the percentage of intersection area of DID districts, the estimation error of this model will be smaller.

Comparison with ESA CCI Land Cover time-series v2.0.7

As a product of the conventional method, urban area of ESA CCI Land Cover time-series v2.0.7 (CCI-LC) was compared with the DID area. We used 2015 land cover map of CCI-LC. Figure S4 shows both areas on the map. Blue indicates the grid in which both CCI-LC and DID are urban areas, green indicates the grid in which only CCI-LC is urban, and red indicates the grid in which only DID is urban. The urban area of CCI-LC is more than 1.5 times that of DID, which indicates that the urban area is overestimated relative to the definition in Japanese urban planning. CCI-LC classifies 22 types of surface coverage, including urban.

The product was created combining various sources. Urban area of CCI-LC relies on Global Human Settlement Layer (Pesaresi et al., 2016) and on the Global Urban Footprint (Esch et al., 2013). Former was based on Landsat images and other spatial data sources to make supervised classification of urban area. Latter used synthetic aperture radar (SAR) images obtained in the TerraSAR-X add-on for Digital Elevation Measurement (TanDEM-X) mission and the urban footprint was obtained by unsupervised classification. Of course these products were not calibrated to fit with the DID; however, the present study is limited to a comparison of our estimation with existing products.

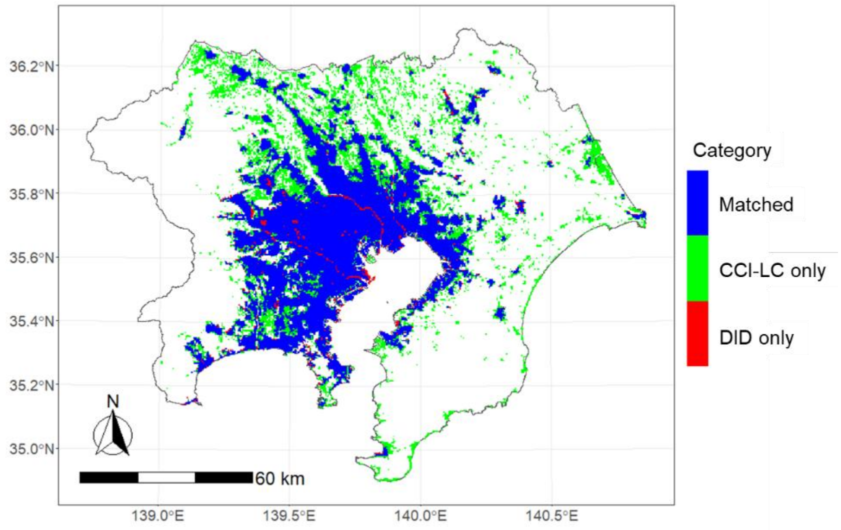

Fig. S4. Conformity of the urban area in CCI-LC to the DID.

Pesaresi, M, Ehrlich D, Ferri S, Florczyk A, Carneiro Freire S, Halkia S, Julea A, Kemper T, Soille P, Syrris V. Operating procedure for the production of the Global Human Settlement Layer from Landsat data of the epochs 1975, 1990, 2000, and 2014. EUR 27741. Luxembourg (Luxembourg): Publications Office of the European Union; 2016. JRC97705

Esch, T. et al. Urban Footprint Processor—Fully Automated Processing Chain Generating Settlement Masks From Global Data of the TanDEM-X Mission. IEEE Geoscience and Remote Sensing Letters 10, 1617-1621 (2013). https://doi.org:10.1109/LGRS.2013.2272953
